# Tópicos Emergentes em Insuficiência Cardíaca: Novos Paradigmas na Amiloidose Cardíaca

**DOI:** 10.36660/abc.20201126

**Published:** 2020-11-01

**Authors:** Marcus Vinicius Simões, Silvia Marinho Martins Alves, Fabio Fernandes, Otávio Rizzi Coelho, Sandrigo Mangini

**Affiliations:** 1 Universidade de São Paulo Faculdade de Medicina de Ribeirão Preto Divisão de Cardiologia São PauloSP Brasil Divisão de Cardiologia, Faculdade de Medicina de Ribeirão Preto, Universidade de São Paulo, São Paulo, SP – Brasil; 2 Universidade de Pernambuco RecifePE Brasil Pronto Socorro Cardiológico de Pernambuco (PROCAPE) - Universidade de Pernambuco, Recife, PE - Brasil; 3 Universidade de São Paulo Faculdade de Medicina Incor – Instituto do Coração (Incor) São PauloSP Brasil Incor – Instituto do Coração (Incor), Faculdade de Medicina, Universidade de São Paulo, São Paulo, SP - Brasil; 4 Universidade Estadual de Campinas Faculdade de Ciências Médicas CampinasSP Brasil Faculdade de Ciências Médicas, Universidade Estadual de Campinas (Unicamp), Campinas, SP - Brasil

**Keywords:** Insuficiência Cardíaca, Miocardiopatia Restritiva, Amiloidose, Imagem Cardiovascular, Doença Cardiovascular

## Abstract

Evidências recentes sugerem que a amiloidose cardíaca é uma doença amplamente subdiagnosticada, particularmente na sua forma ligada à transtirretina, podendo ser uma causa comum de insuficiência cardíaca com fração de ejeção preservada (ICFEP) no idoso. Os novos paradigmas sobre a doença incluem o desenvolvimento de novas terapias específicas que modificam a história natural da doença. Este artigo traz uma síntese destes novos conceitos.

## Mudança de paradigmas da epidemiologia da doença

A amiloidose é uma doença multisistêmica causada pela deposição tecidual de proteínas fibrilares insolúveis que perdem sua conformação, levando à disfunção orgânica, inclusive do coração. Mais de 30 tipos de proteínas amiloidogênicas são descritas,^1^ sendo duas delas responsáveis por 95% dos casos de acometimento cardíaco: a amiloidose por cadeia leve (AL), relacionada à produção monoclonal de imunoglobulinas devido à discrasia de plasmócitos; e a amiloidose por transtirretina (ATTR), proteína transportadora de retinol e tiroxina produzida pelo fígado, podendo ser secundária a sua mutação (ATTRm) ou selvagem (ATTRwt), causada por alterações pós-transcricionais e de chaperonas, ligadas ao envelhecimento.

A AL apresenta incidência em 6 a 10 casos por milhão de pessoas/ano^2^ e era considerada a principal causa de amiloidose cardíaca (AC). No entanto, com o desenvolvimento de técnicas não invasivas de diagnóstico e com o surgimento de tratamentos efetivos, o diagnóstico da ATTR, especialmente da ATTRwt, tem aumentado significativamente.^3^ Estudos demonstram ATTR em até 13%^4^ dos pacientes com fração de ejeção preservada (ICFEP) e espessamento da parede ventricular esquerda > 12 mm, sendo que até 25%^5^ das necropsias de muito idosos apresentam TTR no coração. A ATTRm tem caráter autossômico dominante, com mais de 130 mutações descritas; dependendo de cada mutação, os fenótipos de acometimento neurológico e cardíaco variam.

## Quando suspeitar de amiloidose?

Tendo em vista que a ATTR, particularmente a ATTRwt, é uma condição mais prevalente do que se antecipava, é sempre importante suspeitar dessa condição na presença de pistas clínicas para posterior investigação diagnóstica (Tabela 1).

**Tabela 1:** Pistas diagnósticas para amiloidose cardíaca

**História e exame físico**
ICFEP, particularmente em homens idosos (> 65 anos)
Intolerância aos inibidores da enzima de conversão e ou betabloqueadores
Síndrome do túnel do carpo bilateral
Estenose do canal vertebral
Ruptura do tendão do bíceps
Neuropatia periférica não explicada, particularmente se associada com disfunção autonômica
**Pistas originadas dos exames de imagem**
Captação miocárdica nas imagens cintilográficas com Pirofosfato-Tc99m, grau 2-3
Fenótipo infiltrativo no ecocardiograma, hipertrofia biventricular, derrame pericárdico, espessamento valvar, espessamento de septo interatrial
Redução do *strain* longitudinal que poupa a região apical *(apical sparing)*
Enchimento ventricular esquerdo de padrão restritivo, com espessamento das paredes do ventrículo direito
Realce tardio de contraste na ressonância magnética cardíaca de padrão subendocárdico ou transmural, difuso ou aumento do volume extracelular
**Pistas combinadas**
Insuficiência cardíaca exibindo cavidade ventricular esquerda não dilatada e com aumento do espessamento das paredes
Espessamento concêntrico das paredes do VE com amplitude do QRS desproporcionalmente reduzida ou não aumentada
Função sistólica do VE longitudinal reduzida, apesar da fração de ejeção do ventrículo esquerdo normal
Estenose aórtica com espessamento das paredes do ventrículo direito, particularmente nos casos paradoxais com baixo fluxo/baixo gradiente

Adaptada a partir de Maurer et al. Cic Heart Fail 2019;12:3006075.

Por se tratar de uma forma de cardiomiopatia restritiva infiltrativa, o padrão típico é o espessamento da parede ventricular, a disfunção diastólica e os distúrbios de condução. Em certos contextos clínicos, é necessário o diagnóstico diferencial com cardiomiopatia hipertrófica, ICFEP,^6^ bloqueios atrioventriculares avançados e arritmias atriais sem causa aparente. A concomitância de ATTRwt e estenose aórtica cálcica pode ocasionar hipertrofia ventricular acentuada e pode apresentar-se como estenose aórtica de baixo fluxo e baixo gradiente.

Adicionalmente, certas manifestações multisistêmicas podem levantar suspeita de ATTR: síndrome de tunel do carpo bilateral, ruptura do tendão do bíceps, hipotensão ortostática, estenose do canal vertebral, alterações digestivas e intolerância a medicações anti-hipertensivas.^7^

A história familiar é muito importante nas formas hereditárias da amiloidose, com prognóstico pior do que os pacientes com forma selvagem.

## Métodos diagnósticos

### Eletrocardiograma

A baixa voltagem no complexo QRS é um achado comum na AL, ainda que menos prevalente na ATTR (cerca de 30% dos casos), sendo mais comum a discrepância entre a magnitude da hipertrofia ao ecocardiograma e a amplitude dos complexos QRS. Fibrilação atrial e o padrão de pseudoinfarto também podem ser encontrados.

### Ecocardiograma

É um dos principais exames para levantar a suspeita. Entre os achados sugestivos, destacam-se o espessamento da parede ventricular esquerda > 12 mm, especialmente na ausência de hipertensão arterial, aumento bi-atrial e desproporcional ao tamanho dos ventrículos, espessamento das valvas atrioventriculares e do septo interatrial e aumento da ecogenicidade do miocárdio com aparência granular.^8^

O índice de deformação sistólica longitudinal do miocárdio ou *strain* sistólico longitudinal pode mostrar a preservação da contratilidade do ápice do ventrículo esquerdo (VE) em relação aos demais segmentos (*apical* sparing ou imagem de “cereja do bolo”).^8^

### Cintilografia cardíaca com radiotraçadores ósseos

Cintilografia cardíaca com radiotraçadores ósseos, como Tc99m-Pirofosfato usado no Brasil, pode ser utilizada para o diagnóstico diferencial entre a amiloidose AL e ATTR, esta última mostrando captação miocárdica anômala com intensidade maior ou equivalente à óssea. Contudo, pode ocorrer captação cardíaca, ainda que mais discreta, em até 30% dos casos de AL. Captação cardíaca intensa (grau 2 ou 3), em conjunto com ausência de cadeias leves nos exames bioquímicos, tem especificidade de 100% para ATTR, podendo dispensar a biópsia cardíaca para o diagnóstico da doença.^3^

### Ressonância magnética cardíaca (RMC)

A RMC tem alta sensibilidade e especificidade para o diagnóstico, sendo também útil para diferenciar a AC de outras miocardiopatias. A deposição amiloide no miocárdio causa aumento do volume de distribuição do contraste paramagnético nas regiões do miocárdio, onde os cardiomiócitos são substituídos ou deslocados por fibrose ou inflamação, cursando com padrão de realce tardio (RT) mais comumente subendocárdico difuso e circunferencial do VE, ainda que RT transmural e difuso também possa ser encontrado.^8^

### Abordagem racional para o diagnóstico

**Figura 1:**
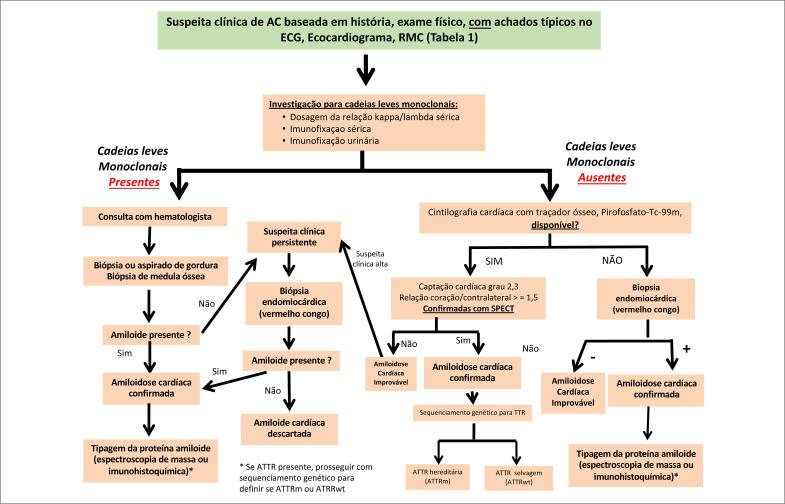
Fluxograma para o diagnóstico da amiloidose cardíaca e suas formas. AC: amiloidose cardíaca; ECG: eletrocardiograma; RMC: ressonância magnética cardíaca; TTR: transtirretina; ATTRm: forma variante da ATTR; TTRwt: forma selvagem da ATTR. Adaptada a partir de Maurer et al. Cic Heart Fail 2019;12:3006075.

Um fluxograma para o diagnóstico da AC é apresentado na Figura 1. Destaca-se que, frente à suspeita da doença (Tabela 1), o primeiro passo é a investigação da presença de cadeias leves para o diagnóstico da AL, uma vez que essa forma da AC exibe tratamento específico com quimioterápicos e o prognóstico piora muito com o retardo no início do tratamento. A confirmação da AL depende da detecção de substância amiloide em tecidos envolvidos (biópsia), mas a forma ATTR pode ser confirmada não invasivamente, mediante emprego de cintilografia cardíaca com Tc-99m-pirofosfato.

### Novas terapias da ATTR

Várias etapas do processo de formação das fibrilas amiloides constituem alvos terapêuticos na ATTR. O estabilizador dos tetrâmeros, tafamidis, foi avaliado em estudo clínico multicêntrico, randomizado e placebo-controlado (estudo ATTR-ACT).^9^ O uso de tafamidis associou-se à redução de 30% na mortalidade por qualquer causa [RR = 0,70 (IC95%: 0,51–0,96)], 32% na redução de internações cardiovasculares [RR = 0,68 (IC95%: 0,56–0,81)] e redução da piora da capacidade funcional e da qualidade de vida. Esses resultados embasaram, no Brasil, a aprovação pela Anvisa do uso de tafamidis para tratamento da AC-ATTR.

Terapias baseadas no silenciamento da expressão dos genes que codificam a produção hepática de TTR são muito promissoras, incluindo estratégias com RNA de interferência (patisiran) e oligonucleotídeos *anti-sensing* (inotersen). Ambas as drogas se mostraram efetivas em reduzir a progressão das manifestações neurológicas e atualmente estão sendo testadas, em estudos multicêntricos. em pacientes com AC-ATTR.^10^^,^^11^

### Lista de Participantes do Heart Failure Summit Brazil 2020 / Departamento de Insuficiência Cardíaca - DEIC/SBC

Aguinaldo Freitas Junior, Andréia Biolo, Antonio Carlos Pereira Barretto, Antônio Lagoeiro Jorge, Bruno Biselli, Carlos Eduardo Montenegro, Denilson Campos de Albuquerque, Dirceu Rodrigues de Almeida, Edimar Alcides Bocchi, Edval Gomes dos Santos Júnior, Estêvão Lanna Figueiredo, Evandro Tinoco Mesquita, Fabiana G. Marcondes-Braga, Fábio Fernandes, Fabio Serra Silveira, Felix José Alvarez Ramires, Fernando Atik, Fernando Bacal, Flávio de Souza Brito, Germano Emilio Conceição Souza, Gustavo Calado de Aguiar Ribeiro, Humberto Villacorta Jr., Jefferson Luis Vieira, João David de Souza Neto, João Manoel Rossi Neto, José Albuquerque de Figueiredo Neto, Lídia Ana Zytynski Moura, Livia Adams Goldraich, Luís Beck-da-Silva, Luís Eduardo Paim Rohde, Luiz Claudio Danzmann, Manoel Fernandes Canesin, Marcelo Bittencourt, Marcelo Westerlund Montera, Marcely Gimenes Bonatto, Marcus Vinicius Simões, Maria da Consolação Vieira Moreira, Miguel Morita Fernandes da Silva, Monica Samuel Avila, Mucio Tavares de Oliveira Junior, Nadine Clausell, Odilson Marcos Silvestre, Otavio Rizzi Coelho Filho, Pedro Vellosa Schwartzmann, Reinaldo Bulgarelli Bestetti, Ricardo Mourilhe Rocha, Sabrina Bernadez Pereira, Salvador Rassi, Sandrigo Mangini, Silvia Marinho Martins, Silvia Moreira Ayub Ferreira, Victor Sarli Issa.
