# Supplementary material for: Tópicos Emergentes em Insuficiência Cardíaca: Novos Paradigmas na Amiloidose Cardíaca
Source: Arq Bras Cardiol. 2020 Nov 1;115(5):945–8. [Article in Portuguese] doi: 10.36660/abc.20201126 (PMC8452217; doi:10.36660/abc.20201126)
Supplement: Translation

# Translation: Emerging Topics in Heart Failure: New Paradigms in Cardiac Amyloidosis

**Authors:** Marcus Vinicius Simões, Silvia Marinho Martins Alves, Fabio Fernandes, Otávio Rizzi Coelho, Sandrigo Mangini

**Affiliations:** 1 São PauloSP Brazil Divisão de Cardiologia, Faculdade de Medicina de Ribeirão Preto, Universidade de São Paulo, São Paulo, SP – Brazil; 2 RecifePE Brazil Pronto Socorro Cardiológico de Pernambuco (PROCAPE) - Universidade de Pernambuco, Recife, PE - Brazil; 3 São PauloSP Brazil Incor – Instituto do Coração (Incor), Faculdade de Medicina, Universidade de São Paulo, São Paulo, SP - Brazil; 4 CampinasSP Brazil Faculdade de Ciências Médicas, Universidade Estadual de Campinas (Unicamp), Campinas, SP - Brazil

**Keywords:** Heart Failure, Restrictive Cardiomyopathy, Amyloidosis, Cardiovascular Imaging, Cardiovascular Disease

## Abstract

Recent evidence suggests cardiac amyloidosis (CA) is a mostly underdiagnosed condition, particularly in the transthyretin-mediated form, and is a frequent cause of heart failure with preserved ejection fraction (HFpEF) in the elderly. New paradigms about CA also involve the development of disease-modifying specific therapies. This article summarizes these new concepts.

## A paradigm shift in amyloidosis epidemiology

Amyloidosis is a multiorgan disease caused by tissue deposition of misfolded insoluble protein fibrils (i.e., that have lost their original conformation), leading to organ dysfunction, including the heart. Although more than 30 types of amyloidogenic proteins have been described,^1^ two types account for to 95% of all cases involving the heart: immunoglobulin light chain (AL), which is related to production of monoclonal immunoglobulins due to a plasma-cell dyscrasia and causes light-chain amyloidosis, and transthyretin, a retinol and thyroxin carrier protein produced in the liver. Transthyretin-mediated amyloidosis (ATTR) can be secondary to an abnormal (mutant or variant) protein (ATTRm) or to the wild-type form (ATTRwt), caused by post-transcriptional modification or by chaperone-related mechanisms – both linked to senescence.

AL has an estimated incidence of 6 to 10 cases per million persons per year,^2^ and was once considered the main cause of CA. However, with the advancement of non-invasive diagnostic methods and the development of effective treatment options, the diagnosis of ATTR – mainly the ATTRw form – is steadily growing.^3^ ATTR is reported in up to 13%^4^ of patients with HFpEF and left ventricular wall thickness >12 mm, and in up to 25%^5^ of hearts in autopsies of the very elderly. ATTRm has an autosomal dominant inheritance pattern; more than 130 mutations have been reported, and the phenotype expression – cardiac or neurologic – varies according to the mutation.

## When to suspect cardiac amyloidosis

Considering that ATTR is more prevalent than previously thought, particularly in the wild-type form, and may masquerade as other common clinical conditions, it is important to adopt a high level of clinical suspicion, including the search for clues that may lead to diagnostic investigation (Table 1).

**Table 1:** Diagnostic clues to cardiac amyloidosis

**Clinical history and physical examination**
HFpEF, particularly in elderly men (age > 65 years)
Angiotensin-converting enzyme inhibitor or beta-blocker intolerance
Bilateral carpal tunnel syndrome
Lumbar canal stenosis
Biceps tendon rupture
Unexplained peripheral neuropathy, particularly when associated with autonomic dysfunction
**Cardiac imaging**
Scintigraphy showing anomalous grade 2-3 increased cardiac uptake of pyrophosphate-Tc99m
Infiltrative phenotype on echocardiogram, with biventricular hypertrophy, pericardial effusion, valve thickening, and interatrial septum thickening
Longitudinal strain rate reduction that spares the apical region (“*apical sparing*” pattern)
Restrictive abnormality of ventricular filling with right ventricular wall thickening
CMR showing late gadolinium enhancement with diffuse subendocardial or transmural pattern, increased ECV
**Combined clues**
Heart failure with unexplained left ventricular wall thickening and a non-dilated ventricular cavity
Concentric left ventricular hypertrophy with reduced or non-increased QRS amplitude
Reduced left ventricular systolic function despite normal global ejection fraction
Aortic stenosis with right ventricular wall thickening, particularly with a paradoxical low flow-low gradient pattern

Adapted from Maurer et al. Cic Heart Fail 2019;12:3006075.

CA is a restrictive infiltrative cardiomyopathy, and the typical presentation involves ventricular wall thickening, diastolic dysfunction, and conduction abnormalities. In given clinical sets, CA should be differentiated from hypertrophic cardiomyopathy, HFpEF,^6^ advanced AV block, and atrial arrhythmias without apparent causes. The simultaneous finding of ATTRwt and calcific aortic stenosis may originate severe left ventricular hypertrophy and may present as paradoxical low-flow and low-gradient aortic stenosis.

Additionally, several systemic manifestations may rouse suspicion of ATTR: bilateral carpal tunnel syndrome, biceps tendon rupture, lumbar canal stenosis, orthostatic hypotension, digestive manifestations, and intolerance to antihypertensive medications.^7^ The family history is very important in the hereditary forms of amyloidosis, which carry a worse prognosis as compared to ATTRwt.

## Diagnostic methods

### Electrocardiography

A low-amplitude QRS complex is a frequent sign in AL, but is quite less prevalent in ATTR (around 30%), which more commonly presents with a discrepancy between the magnitude of left ventricular hypertrophy on the echocardiogram and the QRS voltage. Atrial fibrillation and a “pseudoinfarction” pattern can also be found.

### Echocardiogram

Echocardiography is the most important imaging modality to raise suspicion of CA. Suggestive findings include: left ventricular wall thickness > 12 mm, especially in the absence of arterial hypertension; bi-atrial enlargement disproportional to the dimensions of the ventricular cavities; atrioventricular valve leaflet and atrial septal thickening; and increased myocardial echogenicity with a granular aspect.^8^ Longitudinal strain rate imaging may show the typical pattern of “apical sparing” as compared to reduced contractility in the remaining segments.^8^

### Cardiac scintigraphy with bone-avid radiotracers

Cardiac scintigraphy with bone-avid radiotracers, such as technetium Tc99m pyrophosphate as used in Brazil, may be employed to distinguish AL from ATTR, with the latter showing anomalous myocardial uptake higher than the uptake observed in the ribs. However, cardiac uptake may occur, albeit with milder intensity, in up to 30% of AL cases. The combination of intense cardiac uptake (grades 2 or 3) and negative biochemical investigation for monoclonal light chains is 100% specific for ATTR, and can obviate endomyocardial biopsy for diagnosis.^3^

### Cardiac magnetic resonance

Cardiac magnetic resonance (CMR) imaging has high sensitivity and specificity for CA diagnosis, while also allowing identification of other myocardial diseases. Amyloid deposits in the myocardium increase the distribution volume of the paramagnetic contrast agent in myocardial regions where the cardiomyocytes are displaced by the deposits, inflammation, or fibrosis, originating a diffuse subendocardial and circumferential late enhancement pattern; a diffuse transmural pattern can also be found.^8^

### Rational diagnostic approach

Figure 1 illustrates a proposed diagnostic algorithm for CA. It highlights that, when CA is suspected (table 1), the first step should consist of a monoclonal light chain assay with a view to AL diagnosis, as specific chemotherapeutic management is available for this form of CA and the prognosis worsens dramatically if treatment onset is delayed. Confirmation of AL relies on detection of the amyloid protein in the involved organ tissue through biopsy, but the ATTR form can be diagnosed non-invasively by cardiac scintigraphy with technetium-Tc99m pyrophosphate as described above.

**Figure 1:** Algorithm for CA diagnosis

### New therapies for ATTR

Several steps of amyloid fiber formation in ATTR constitute therapeutic targets. The tetramer stabilizer tafamidis was evaluated in a multicenter, randomized, placebo-controlled trial (ATTR-ACT study).^9^ Tafamidis was associated with a 30% reduction in all-cause mortality (RR=0.70, 95%CI 0.51–0.96), a 32% reduction in cardiovascular hospitalization (RR=0.68, 95%CI 0.56–0.81) and reduction of the rate of deterioration of functional capacity and quality of life. Based on these results, tafamidis was approved by ANVISA for the treatment of ATTR-CA.

Therapies based on silencing the expression of genes that codify the hepatic production of TTR are very promising, including small interference RNA (patisiran) and antisense oligonucleotides (inotersen). Both strategies have proven effective in reducing the progression of neurologic manifestations in ATTR and are currently under evaluation in multicenter studies for the treatment of ATTR-CA.^10^^,^^11^

### List of participants of the Heart Failure Summit Brazil 2020 / Heart Failure Department - Brazilian Society of Cardiology

Aguinaldo Freitas Junior, Andréia Biolo, Antonio Carlos Pereira Barretto, Antônio Lagoeiro Jorge, Bruno Biselli, Carlos Eduardo Montenegro, Denilson Campos de Albuquerque, Dirceu Rodrigues de Almeida, Edimar Alcides Bocchi, Edval Gomes dos Santos Júnior, Estêvão Lanna Figueiredo, Evandro Tinoco Mesquita, Fabiana G. Marcondes-Braga, Fábio Fernandes, Fabio Serra Silveira, Felix José Alvarez Ramires, Fernando Atik, Fernando Bacal, Flávio de Souza Brito, Germano Emilio Conceição Souza, Gustavo Calado de Aguiar Ribeiro, Humberto Villacorta Jr., Jefferson Luis Vieira, João David de Souza Neto, João Manoel Rossi Neto, José Albuquerque de Figueiredo Neto, Lídia Ana Zytynski Moura, Livia Adams Goldraich, Luís Beck-da-Silva Neto, Luís Eduardo Paim Rohde, Luiz Claudio Danzmann, Manoel Fernandes Canesin, Marcelo Bittencourt, Marcelo Westerlund Montera, Marcely Gimenes Bonatto, Marcus Vinicius Simões, Maria da Consolação Vieira Moreira, Miguel Morita Fernandes da Silva, Monica Samuel Avila, Mucio Tavares de Oliveira Junior, Nadine Clausell, Odilson Marcos Silvestre, Otavio Rizzi Coelho Filho, Pedro Vellosa Schwartzmann, Reinaldo Bulgarelli Bestetti, Ricardo Mourilhe Rocha, Sabrina Bernadez Pereira, Salvador Rassi, Sandrigo Mangini, Silvia Marinho Martins, Silvia Moreira Ayub Ferreira, Victor Sarli Issa.
